# Machine Learning–Based Prediction of Disease-Free Survival in Breast Cancer Incorporating Clinical and Treatment Information: Retrospective Cohort Study With External Validation

**DOI:** 10.2196/101633

**Published:** 2026-11-13

**Authors:** Zhifu Chen, Yuxuan Wu, Li Tong, Ting Wu, Nan Zhao, Lihua Zhu, Yiyao Zhu, Yan Wang, Rui Shao, Yishan Huang

**Affiliations:** 1 Department of Pathology, The Affiliated People's Hospital of Jiangsu University, No. 8 Dianli Road, Runzhou District, Zhenjiang, Jiangsu, 212000, China, 86 88915601; 2 Department of Medical Records, The Affiliated People's Hospital of Jiangsu University, Zhenjiang, Jiangsu, China; 3 Department of Radiation Oncology, The Affiliated People's Hospital of Jiangsu University, Zhenjiang, Jiangsu, China; 4 Neuroscience Center, Shantou University Medical College, Shantou, Guangdong, China

**Keywords:** breast cancer, recurrence prediction, machine learning, clinicopathological indicators, prognostic model, Shapley additive explanations, SHAP

## Abstract

**Background:**

Breast cancer is the most prevalent cancer in women, and postoperative disease-free survival (DFS) is an important indicator of long-term prognosis. However, tumor, node, metastasis staging has limited ability to integrate multidimensional clinical, pathological, and treatment-related factors for individualized DFS prediction. Therefore, machine learning (ML) may provide a more comprehensive approach to prognostic risk assessment.

**Objective:**

This study aimed to develop and externally validate a ML-based model for predicting postoperative DFS in patients with breast cancer.

**Methods:**

This retrospective study included 2 cohorts: 1231 patients treated at an institutional center between 2012 and 2020 and 304 patients from The Cancer Genome Atlas (TCGA). The institutional cohort was divided into model development (n=861) and internal validation (n=370) sets, while the TCGA cohort served as an external validation cohort. Five ML algorithms were evaluated, with the Cox proportional hazards model additionally fitted as a conventional statistical reference. Model performance was assessed using the concordance index and time-dependent area under the curve (AUC). The net reclassification index and integrated discrimination improvement were used for comparative evaluation. Shapley additive explanations (SHAP) analysis was performed to assess predictor contributions, and patients were stratified into risk groups according to the median predicted risk score.

**Results:**

Based on a comprehensive evaluation of model performance, extreme gradient boosting (XGBoost) with an accelerated failure time (AFT) was selected as the final model. The study included 1231 institutional patients, with 861 used for model development and 370 for internal validation, and 304 patients from TCGA for external validation. The 5-year time-dependent AUCs were 0.840, 0.822, and 0.762 in the training, internal validation, and external validation cohorts, respectively. In the external validation cohort, the XGBoost (AFT) model achieved a concordance index of 0.750 (95% CI 0.654‐0.843), with 3-, 5-, and 8-year AUCs of 0.791, 0.762, and 0.768, respectively. At 5 years, compared with Cox regression, XGBoost (AFT) showed improved reclassification (net reclassification index=0.311, 95% CI 0.172‐0.458; *P*=.01; integrated discrimination improvement=0.166, 95% CI 0.095‐0.235; *P*<.001). SHAP analysis identified endocrine therapy, pT, pN, lymph node ratio, Ki-67, and chemotherapy among the major contributors to model predictions. Risk stratification based on the median predicted risk score separated patients into groups with distinct DFS outcomes (hazard ratio 5.44, 95% CI 4.04‐7.31).

**Conclusions:**

The ML-based model demonstrated favorable performance and external generalizability in predicting postoperative DFS. SHAP analysis provided interpretability for the model, and risk stratification identified patient subgroups with distinct prognostic risks.

## Introduction

Breast cancer is the most prevalent malignancy worldwide, with the latest data indicating approximately 2.3 million new breast cancer diagnoses annually, making it a leading cause of cancer-related mortality among women [1]. Although the overall survival rate of patients with breast cancer has significantly improved due to the widespread adoption of early screening technologies and advancements in comprehensive treatment plans, including surgery, chemotherapy, radiotherapy, and endocrine therapy, postoperative disease-free survival (DFS) remains a severe challenge for long-term survival [2]. It has been reported that approximately 20% to 30% of patients with breast cancer experience local DFS or distant metastasis within 5 to 10 years after diagnosis, which severely compromises patient survival prognosis and quality of life and imposes a substantial burden on health care systems and society [3]. Therefore, the accurate assessment of DFS risk in patients with breast cancer has important clinical implications. It can provide an intensified therapy protocol for high-risk patients and guide the formulation of individualized adjuvant treatment plans and follow-up strategies.

Currently, the clinical assessment of breast cancer DFS prognosis traditionally relies on the tumor, node, metastasis (TNM) staging system established by the American Joint Committee on Cancer [4]. However, breast cancer exhibits high heterogeneity, implying that patients with similar clinical stages and pathological characteristics can have markedly different clinical outcomes [5]. Although these factors have been incorporated into classic prognostic models such as the Nottingham Prognostic Index, the predictive performance is limited by the heterogeneity of breast cancer and the confounding relationships among multiple prognostic variables [6]. Furthermore, with the development of high-throughput technologies and the availability of multiomics data such as gene expression, DNA methylation, and proteomics, traditional statistical models (eg, Cox proportional hazards regression) demonstrate certain limitations in analyzing complex, nonlinear interactions within clinical data [7].

In recent years, machine learning (ML) algorithms have emerged as powerful tools for processing high-dimensional, nonlinear, and heterogeneous data, offering novel approaches to enhance the accuracy and robustness of DFS prediction models [8]. Unlike traditional statistical methods that require explicit assumptions about the data distribution and variable relationships, ML algorithms can automatically learn complex patterns and latent associations from large-scale datasets through iterative training and optimization [9]. Previous studies have shown that ML models integrating clinicopathological features, multiomics data, or imaging data outperform traditional prognostic models in predicting breast cancer DFS [10]. Additionally, ML models can provide interpretable results through feature importance analysis, aiding in the identification of novel biomarkers and offering new prognostic indicators for clinical practice.

Despite the advantages and general applicability of ML models over traditional prediction methods, their clinical application can still lead to biased results due to inadequate dataset size and training data [11]. Therefore, it is essential to develop robust and interpretable ML models for breast cancer DFS prediction using large-scale sample sets, followed by rigorous external validation and clinical correlation assessment. This study aimed to develop a DFS prediction model that integrates clinicopathological features from The Cancer Genome Atlas (TCGA) and an independent validation cohort to predict breast cancer DFS risk and evaluate its performance relative to traditional prognostic models and existing ML models.

## Methods

### Data Source and Patients Selection

A total of 1869 female patients diagnosed with breast cancer were retrospectively screened from January 2012 to December 2020 at the Affiliated People’s Hospital of Jiangsu University. Patients were eligible if they met the following criteria: age greater than 18 years, newly diagnosed T1-4 N0-3 M0 breast cancer confirmed by pathological diagnosis, and receipt of surgical treatment. Patients who were lost to follow-up, aged more than 75 years, had distant metastasis at initial diagnosis, or had an uncertain diagnosis were excluded. Finally, 1231 patients were included in the institutional cohort.

The 1231 patients in the institutional cohort were randomly divided into a training cohort (n=861) and an internal validation cohort (n=370) at a ratio of 7:3. The training cohort was used for feature selection, model development, and hyperparameter optimization, whereas the internal validation cohort was used to evaluate model performance in previously unseen patients. The difference in sample size between the training and internal validation cohorts was a direct consequence of the prespecified 7:3 random partitioning strategy.

For external validation, data from 1097 patients were independently obtained from TCGA database, including clinical information and RNA sequencing profiles. Applying the same eligibility criteria, 304 patients were included in the external validation cohort. The TCGA cohort was completely independent of the institutional cohort and was not used for feature selection, model development, or hyperparameter optimization.

Missing data in the TCGA cohort were handled using multiple imputation with the “mice” package in R [12]. For the continuous variable lymph node ratio (LNR; 27/304 missing), predictive mean matching was applied. Binary variables, including menopausal status (28/304 missing), chemotherapy, endocrine therapy, and targeted therapy (32/304 missing), radiotherapy (19/304 missing), and estrogen receptor (ER) status (1/304 missing), were imputed using logistic regression.

In the institutional cohort, Ki-67 expression was assessed by immunohistochemistry and categorized as low (<20%) or high (≥20%), based on the 2013 St. Gallen International Expert Consensus and the 2018 guidelines [13,14]. This 20% cutoff was selected to reflect contemporary clinical practice and consensus-based assessment during the study period. Because standardized Ki-67 and P53 immunohistochemical measurements were not available in the TCGA-BRCA (The Cancer Genome Atlas Breast Invasive Carcinoma) cohort, RNA sequencing data were used as transcriptomic surrogate measures, with MKI67 and TP53 expression corresponding to Ki-67 and P53, respectively. For external validation, the MKI67 and TP53 expression values were dichotomized using thresholds derived to approximate the corresponding distribution in the institutional cohort based on the 25th percentile (lower quartile) of expression. These transcriptomic measures were used as validation surrogates and were not considered directly equivalent to immunohistochemistry-derived Ki-67 and P53 measurements in the institutional cohort. DFS was defined as the time from the date of surgery to the first occurrence of disease recurrence, metastasis, or death from any cause. Patients without any of these events were censored at the date of their last available follow-up.

The candidate predictors included demographic, pathological, perioperative, and postoperative treatment variables. Demographic and pathological variables included age, menopausal status, LNR, tumor laterality, pT stage, pN stage, ER status, progesterone receptor status, and human epidermal growth factor receptor 2 (HER2) status. Surgery type was considered a perioperative clinical variable. Chemotherapy, endocrine therapy, targeted therapy, and radiotherapy represented postoperative treatments actually received by the patients rather than treatment plans available at the time of surgery. These treatment variables were incorporated to develop a postoperative, treatment-informed DFS prediction model. Their predictive importance was interpreted as prognostic information contained in the observed postoperative treatment history and not as evidence of a causal treatment effect.

### Model Building and Evaluation

The 1231 patients with breast cancer were randomly divided into a training cohort and an internal validation cohort at a ratio of 7:3. In addition, 304 patients with breast cancer screened from the TCGA database were included as an external validation cohort. Five ML methods, including random survival forest, survival support vector machine, CoxBoost, light gradient boosting machine (LightGBM), and extreme gradient boosting (XGBoost) with an accelerated failure time (AFT) objective, together with Cox regression, were used to develop prediction models. Harrell’s C-index, the area under the curve (AUC) at 3, 5, and 8 years, and the integrated Brier score were used to compare the 6 models. Calibration curves at 3, 5, and 8 years and decision curve analysis were used to evaluate model calibration and clinical utility. In the calibration curves, the number of bins was adaptively adjusted based on the sample size of each cohort to ensure the stability of the Kaplan-Meier estimates within each group [15]. Bootstrap resampling method (2000 iterations) was applied during model development and validation to enhance the reliability of the study results [16].

### Model Interpretability and Risk Modeling

The Shapley additive explanations (SHAP) method is an advanced ML tool used to interpret the outputs of predictive models. SHAP analysis was performed on the XGBoost (AFT) model to enhance model transparency and facilitate clinical interpretability. The global importance of each variable was quantified using mean absolute SHAP values, and the variables were subsequently ranked. The SHAP force plot was used to interpret individual model predictions. It decomposes the predicted output into contributions from each feature, starting from the base value. Positive and negative SHAP values indicate features that increase or decrease the predicted outcome, respectively. This plot intuitively shows the magnitude and direction of each feature’s effect on individual prediction results.

Variables with a mean absolute SHAP value contribution of 5% were considered to have a nonnegligible contribution to model prediction and were thus selected as candidate predictors. Subsequently, these candidate factors were used to construct a composite DFS risk score model. The risk score for each patient was calculated as a weighted linear combination of the selected variables and their corresponding regression coefficients. The performance of the risk score model was validated using XGBoost (AFT), and the model was applied for risk stratification and further prognostic assessment. The study was reported in accordance with the guidelines (Checklist 1).


$$Risk\;Score=\sum_{i=1}^{N}\left(\beta_{i}\times X_{i}\right),$$

where *N* is the number of prognostic variables, *X_i_* represents the *i*th variable, and *β_i_* is the regression coefficient derived from the Cox regression model.

### Statistical Analysis

The comparison of clinicopathological characteristics between the training and internal validation cohorts was conducted using the Mann-Whitney *U* test, whereas the comparison of clinicopathological characteristics among the training, internal validation, and external validation cohorts was conducted using the Kruskal-Wallis *H* test. Given that all variables included in this study were categorical, intervariable correlations were evaluated using Cramér *V* coefficient to ensure methodological appropriateness and robustness [17].

ML model development was performed using Python (version 3.13.9), incorporating algorithms including XGBoost (AFT), LightGBM, random survival forest, and fast survival support vector machine, implemented via the scikit-survival framework. Detailed hyperparameter settings for each model are provided in Table S1 in Multimedia Appendix 1. All remaining statistical analyses and visualizations were conducted using R (version 4.5.2; R Foundation for Statistical Computing), primarily relying on the survival, tidyverse, ggplot2, tableone, and mclust packages. A 2-tailed *P*<.05 was considered statistically significant.

### Ethical Considerations

This study was conducted in accordance with the Declaration of Helsinki and its subsequent amendments. All data were anonymized, and the requirement for informed consent was waived due to the retrospective nature of the study. The study was reviewed and approved by the Ethics Committee of the Affiliated People’s Hospital of Jiangsu University (approval ID: [2026] KYW001-27) on January 23, 2026. Patients diagnosed between January 2012 and December 2020 were retrospectively identified, and the study protocol was subsequently reviewed and approved by the Ethics Committee. Because this was a retrospective study involving the evaluation of anonymized data, the Ethics Committee of the Affiliated People's Hospital of Jiangsu University waived the requirement for patient consent.

## Results

### Patient Characteristics

In the entire cohort of 1231 patients, the median follow-up duration was 120.6 (IQR 95.1-145.5) months, estimated using the reverse Kaplan-Meier method, and the median DFS was 109.6 (IQR 85.0-134.2) months, based on the conventional Kaplan-Meier analysis. In the external TCGA validation cohort, the median follow-up duration was 49 (IQR 24.8-76.1) months, whereas the median DFS was 42.5 (IQR 24.6-73.6) months. At 5 and 8 years, 122 and 70 patients, respectively, remained at risk in the TCGA cohort (Figure 1).

**Figure 1.**
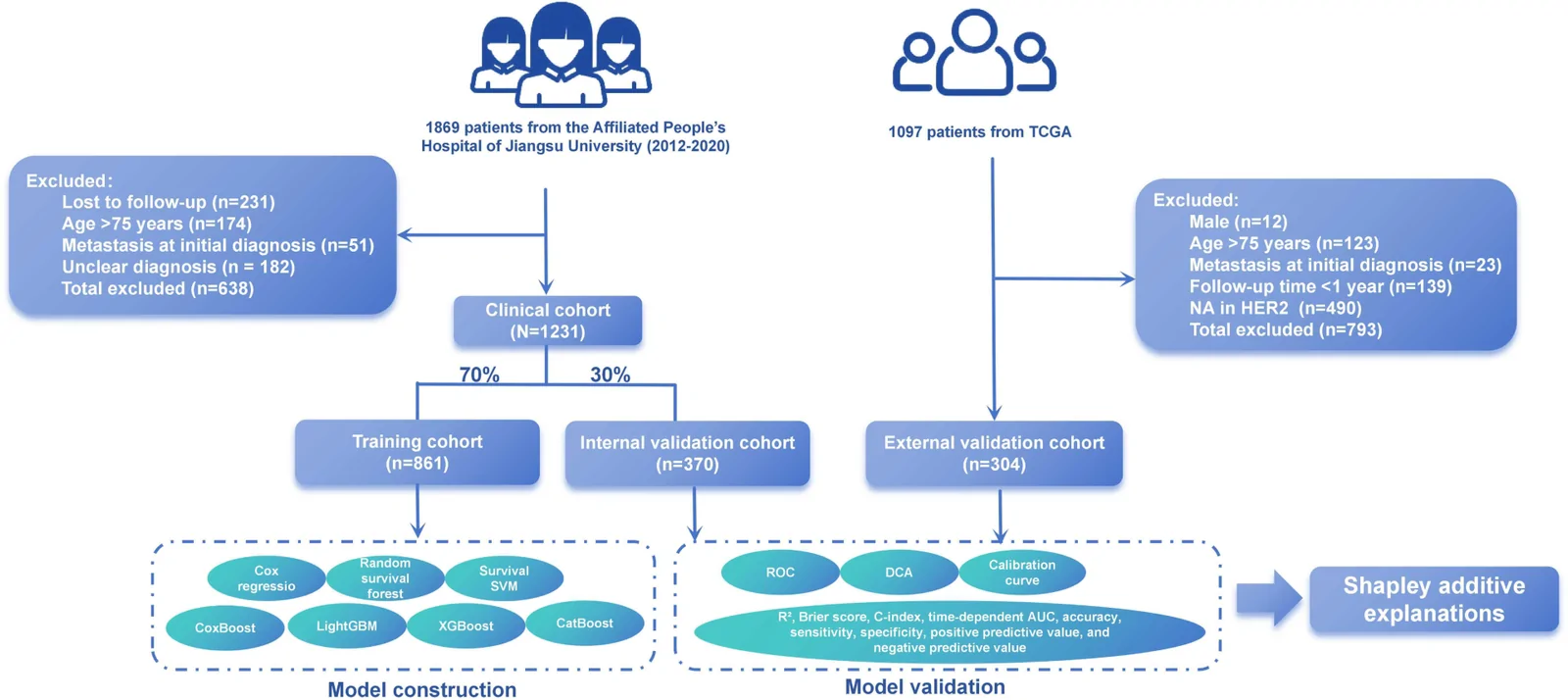
Flowchart of the study design and patient selection. AUC: area under the curve; C-index: concordance index; DCA: decision curve analysis; HER2: human epidermal growth factor receptor 2; LightGBM: light gradient boosting machine; NA: not available; ROC: receiver operating characteristic; SHAP: Shapley additive explanations; SVM: support vector machine; TCGA: The Cancer Genome Atlas; XGBoost: extreme gradient boosting.

The baseline characteristics of patients in the training, internal validation, and external validation cohorts are summarized in Table 1. Across the 3 cohorts, significant differences were observed in most variables, except laterality, reflecting heterogeneity in patient populations and data sources. Nevertheless, the relatively comparable distributions between the training and internal validation cohorts supported model development and internal validation, while the independent external cohort provided an assessment of model generalizability. Importantly, the model maintained satisfactory predictive performance despite the heterogeneity of the external cohort, which is encouraging for its potential clinical applicability. However, these findings should be interpreted cautiously, and further validation in larger, multicenter cohorts is warranted to confirm the robustness and generalizability of the model.

**Table 1.** Baseline clinicopathological characteristics of patients in different cohorts.

Variables	Overall (N=1535), n (%)	Training cohort (n=861), n (%)	Internal validation cohort (n=370), n (%)	*P* value^a^	SMD^a^^,^^b^	External validation cohort (n=304), n (%)	*P* value^c^	SMD^c^
Age (y)				.42	0.054		<.001	0.257
<60	1100 (71.7)	657 (76.3)	265 (71.6)			178 (58.6)		
≥60	435 (28.3)	204 (23.7)	105 (28.4)			126 (41.4)		
Menopausal status				.46	0.050		<.001	0.219
No	888 (57.9)	471 (54.7)	203 (54.9)			214 (70.4)		
Yes	647 (42.1)	390 (45.3)	167 (45.1)			90 (29.6)		
Lymph node ratio				.74	0.092		.009	0.173
<0.2	1140 (74.3)	617 (71.7)	273 (73.8)			250 (82.2)		
0.2-0.65	301 (19.6)	187 (21.7)	75 (20.3)			39 (12.8)		
>0.65	94 (6.1)	57 (6.6)	22 (5.9)			15 (4.9)		
Laterality				.83	0.017		.94	0.017
Right	729 (47.5)	410 (47.6)	173 (46.8)			146 (48.0)		
Left	806 (52.5)	451 (52.4)	197 (53.2)			158 (52.0)		
pT				.76	0.067		.002	0.193
1	510 (33.2)	294 (34.1)	132 (35.7)			84 (27.6)		
2	901 (58.7)	513 (59.6)	210 (56.8)			178 (58.6)		
3	107 (7.0)	46 (5.3)	24 (6.5)			37 (12.2)		
4	17 (1.1)	8 (0.9)	4 (1.1)			5 (1.6)		
pN				.86	0.054		.008	0.192
0	779 (50.7)	442 (51.3)	185 (50)			152 (50)		
1	427 (27.8)	219 (25.4)	101 (27.3)			107 (35.2)		
2	211 (13.7)	133 (15.4)	53 (14.3)			25 (8.2)		
3	118 (7.7)	67 (7.8)	31 (8.4)			20 (6.6)		
ER^d^				.36	0.061		.003	0.156
Negative	466 (30.4)	285 (33.1)	112 (30.3)			69 (22.7)		
Positive	1069 (69.6)	576 (66.9)	258 (69.7)			235 (77.3)		
PR^e^				.33	0.065		.019	0.126
Negative	590 (38.4)	353 (41)	140 (37.8)			97 (31.9)		
Positive	945 (61.6)	508 (59)	230 (62.2)			207 (68.1)		
Ki-67 expression				.93	0.009		.004	0.152
Low	508 (33.1)	301 (35)	131 (35.4)			76 (25)		
High	1027 (66.9)	560 (65)	239 (64.6)			228 (75)		
P53 expression				.41	0.057		<.001	0.251
Low	1310 (85.3)	752 (87.3)	330 (89.2)			228 (75)		
High	225 (14.7)	109 (12.7)	40 (10.8)			76 (25)		
HER2^f^				.84	0.067		.003	0.162
Negative	1133 (73.8)	621 (72.1)	264 (71.4)			248 (81.6)		
Positive	402 (26.2)	240 (27.9)	106 (28.6)			56 (18.4)		
Surgery				.17	0.090		<.001	0.923
Mastectomy	405 (26.4)	127 (14.8)	61 (16.5)			217 (71.4)		
Lumpectomy	1130 (73.6)	734 (85.2)	309 (83.5)			87 (28.6)		
Chemotherapy				.20	0.084		.01	0.127
No	232 (15.1)	112 (13)	59 (15.9)			61 (20.1)		
Yes	1303 (84.9)	749 (87)	311 (84.1)			243 (79.9)		
Endocrine therapy				.67	0.051		.30	0.069
No	550 (35.8)	320 (37.2)	132 (35.7)			98 (32.2)		
Yes	985 (64.2)	541 (62.8)	238 (64.3)			206 (67.8)		
Targeted therapy				.29	0.070		<.001	0.322
No	1212 (79)	653 (75.8)	278 (75.1)			281 (92.4)		
Yes	323 (21)	208 (24.2)	92 (24.9)			23 (7.6)		
Radiation therapy				.27	0.073		<.001	0.479
No	912 (59.4)	565 (65.6)	247 (66.8)			100 (32.9)		
Yes	623 (40.6)	296 (34.4)	123 (33.2)			204 (67.1)		

a The comparison of clinicopathological characteristics between the training and internal validation cohorts was conducted using the Mann-Whitney *U* test.

b SMD: standardized mean differences.

c The comparison of clinicopathological characteristics among the training, internal validation, and external validation cohorts was conducted using the Kruskal-Wallis *H* test.

d ER: estrogen receptor.

e PR: progesterone receptor.

f HER2: human epidermal growth factor receptor 2.

As shown in Figure S1 in Multimedia Appendix 1, most pairwise associations among variables were weak to moderate (Cramér *V*<0.30), with stronger correlations limited to clinically related features. Nevertheless, all covariates included in the multivariable analyses had variance inflation factors <3 (Table S2 in Multimedia Appendix 1), indicating no substantial multicollinearity.

### Model Development and Performance

Multivariable Cox regression analysis (Table 2) identified several variables significantly associated with DFS. Patients who received endocrine therapy had a lower hazard of DFS events than those who did not (hazard ratio [HR] 0.28, 95% CI 0.18‐0.43; *P*<.001). Compared with patients with T1 disease, those with T2, T3, and T4 disease had higher hazards of DFS events. Similarly, compared with N0 disease, N1, N2, and N3 disease were associated with higher hazards of DFS events (HRs 1.68, 2.08, and 2.73, respectively). ER status was also significantly associated with DFS in the multivariable model, with the direction of the estimated association differing from that observed in the univariable analysis after adjustment for other covariates, including postoperative endocrine therapy. Kaplan-Meier curves were subsequently plotted for all variables that demonstrated significance in the univariable analysis (Figure S2 in Multimedia Appendix 1).

**Table 2.** Univariate and multivariate cox regression results for disease-free survival or mortality risk of breast cancer in training cohort.

Variables	Univariate		Multivariate	
	HR^a^ (95% CI)	*P* value	HR (95% CI)	*P* value
Age (y)				
<60	Ref^b^	—^c^	—	—
≥60	1.32 (0.96‐1.82)	.09	—	—
Menopausal status				
No	Ref^b^	—	Ref	—
Yes	0.68 (0.51‐0.91)	.01	0.81 (0.60‐1.10)	.18
Lymph node ratio				
<0.2	Ref^b^	—	Ref^b^	—
0.2‐0.65	3.40 (2.50‐4.63)	<.001	1.63 (0.89‐2.98)	.11
>0.65	4.76 (3.13‐7.22)	<.001	1.56 (0.65‐3.78)	.32
Laterality				
Right	Ref^b^	—	—	—
Left	0.99 (0.74‐1.31)	.92	—	—
pT				
1	Ref^b^	—	Ref^b^	—
2	2.57 (1.74‐3.81)	<.001	1.82 (1.22‐2.72)	.004
3	7.42 (4.42‐12.43)	<.001	3.88 (2.21‐6.79)	<.001
4	13.28 (5.54‐31.86)	<.001	5.72 (2.12‐15.44)	<.001
pN				
0	Ref^b^	—	Ref^b^	—
1	1.93 (1.30‐2.86)	.001	1.68 (1.07‐2.62)	.02
2	4.04 (2.77‐5.90)	<.001	2.08 (1.02‐4.24)	.045
3	5.99 (3.91‐9.16)	<.001	2.73 (1.12‐6.65)	.03
ER^d^				
Negative	Ref^b^	—	Ref^b^	—
Positive	0.70 (0.52‐0.93)	.02	2.31 (1.50‐3.57)	<.001
PR^e^				
Negative	Ref^b^	—	—	—
Positive	0.80 (0.60‐1.06)	.13	—	—
Ki67 expression				
Low	Ref^b^	—	Ref^b^	—
High	1.42 (1.04‐1.94)	.03	1.18 (0.85‐1.62)	.32
P53 expression				
Low	Ref^b^	—	—	—
High	0.90 (0.58‐1.4)	.63	—	—
HER2^f^				
Negative	Ref^b^	—	—	—
Positive	1.23 (0.90‐1.67)	.19	—	—
Surgery				
Mastectomy	Ref^b^	—	Ref^b^	—
Lumpectomy	0.57 (0.40‐0.81)	.002	0.98 (0.68‐1.42)	.92
Chemotherapy				
No	Ref^b^	—	—	—
Yes	0.72 (0.48‐1.07)	.10	—	—
Endocrine therapy				
No	Ref^b^	—	Ref^b^	—
Yes	0.45 (0.34‐0.60)	<.001	0.28 (0.18‐0.43)	<.001
Targeted therapy				
No	Ref^b^	—	—	—
Yes	0.86 (0.61‐1.22)	.40	—	—
Radiation therapy				
No	Ref^b^	—	Ref^b^	—
Yes	1.49 (1.12‐1.99)	.006	0.90 (0.65‐1.25)	.54

a HR: hazard ratio.

b Ref: reference.

c Not applicable.

d ER: estrogen receptor.

e PR: progesterone receptor.

f HER2: human epidermal growth factor receptor.

Table 3 presents the predictive performance of 5 ML models in the development, internal validation, and external validation cohorts, evaluated based on discrimination and overall prediction error. In the development cohort, all models demonstrated good discriminatory performance. The C-indexes of the XGBoost (AFT) and LightGBM models exceeded 0.80. Consistently, their AUC values at 3, 5, and 8 years were also above 0.80 (Figure 2A-C).

**Figure 2.**
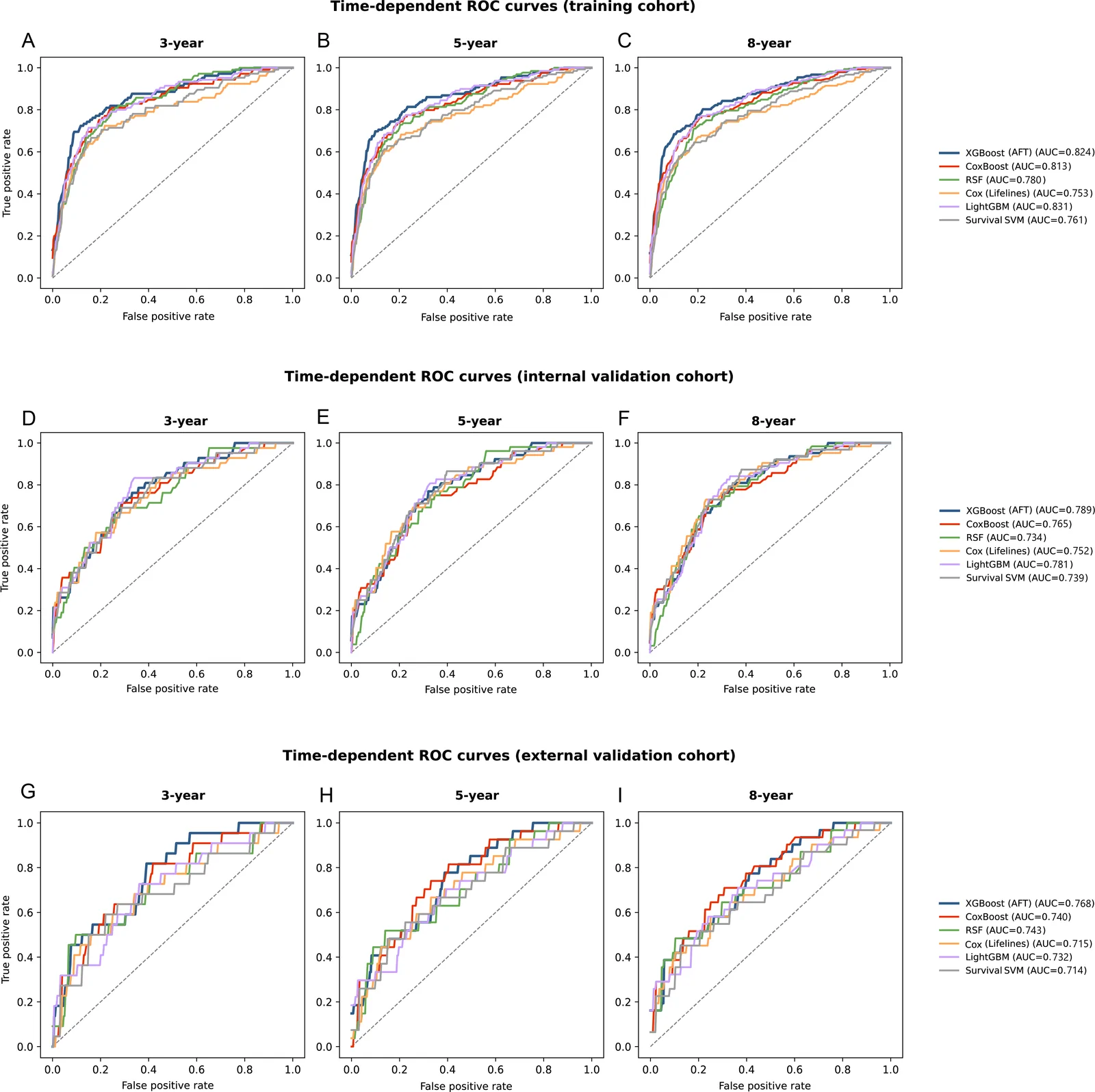
ROC curves of the breast cancer disease-free survival prediction model. (A-C) ROC curves of the five machine learning models and the Cox proportional hazards model in the development set. (D-F) ROC curves of the five machine learning models and the Cox proportional hazards model in the internal validation set. (G-I) ROC curves of the 5 machine learning models and the Cox proportional hazards model in the The Cancer Genome Atlas validation set. AFT: accelerated failure time; AUC: area under the curve; LightGBM: light gradient boosting machine; ROC: receiver operating characteristic; RSF: random survival forest; SVM: support vector machine; XGBoost: extreme gradient boosting.

**Table 3.** Performance comparison of models for predicting the disease-free survival in the training, internal validation, and external validation cohort^a^.

Cohorts and models	Harrell C-index (95% CI)	3-year AUC^b^ (95% CI)	5-year AUC (95% CI)	8-year AUC (95% CI)	Integrated Brier score (95% CI)
Training cohort (n=861)					
Cox regression	0.739 (0.694-0.771)	0.797 (0.722-0.858)	0.766 (0.708-0.807)	0.753 (0.702-0.798)	0.101 (0.091-0.112)
RSF^c^	0.771 (0.732-0.800)	0.807 (0.764-0.845)	0.807 (0.762-0.840)	0.780 (0.735-0.818)	0.103 (0.093-0.114)
Survival SVM^d^	0.750 (0.719-0.782)	0.776 (0.727-0.825)	0.781 (0.743-0.827)	0.761 (0.721-0.807)	0.102 (0.092-0.114)
CoxBoost	0.794 (0.769-0.827)	0.820 (0.779-0.862)	0.825 (0.794-0.866)	0.813 (0.776-0.848)	0.087 (0.075-0.097)
LightGBM	0.805 (0.781-0.839)	0.825 (0.788-0.867)	0.836 (0.801-0.870)	0.831 (0.799-0.864)	0.089 (0.079-0.098)
XGBoost (AFT^e^)	0.808 (0.771-0.832)	0.833 (0.795-0.874)	0.840 (0.796-0.867)	0.824 (0.787-0.856)	0.103 (0.093-0.114)
Internal validation cohort (n=370)					
Cox regression	0.754 (0.701-0.794)	0.759 (0.701-0.809)	0.787 (0.714-0.833)	0.752 (0.693-0.806)	0.103 (0.090-0.119)
RSF	0.752 (0.690-0.792)	0.800 (0.721-0.860)	0.790 (0.711-0.836)	0.734 (0.661-0.788)	0.110 (0.097-0.137)
Survival SVM	0.755 (0.696-0.799)	0.809 (0.747-0.863)	0.794 (0.735-0.849)	0.739 (0.658-0.794)	0.106 (0.090-0.118)
CoxBoost	0.765 (0.713-0.808)	0.811 (0.740-0.862)	0.804 (0.741-0.855)	0.765 (0.741-0.855)	0.103 (0.089-0.121)
LightGBM	0.780 (0.742-0.814)	0.822 (0.751-0.876)	0.827 (0.782-0.869)	0.781 (0.725-0.826)	0.100 (0.087-0.117)
XGBoost (AFT)	0.784 (0.727-0.833)	0.828 (0.773-0.880)	0.822 (0.754-0.875)	0.789 (0.715-0.854)	0.107 (0.093-0.123)
External validation cohort (n=304)					
Cox regression	0.707 (0.584-0.819)	0.717 (0.571-0.834)	0.716 (0.600-0.818)	0.715 (0.551-0.850)	0.055 (0.041-0.069)
RSF	0.728 (0.629-0.819)	0.726 (0.620-0.841)	0.735 (0.602-0.840)	0.743 (0.593-0.853)	0.057 (0.039-0.073)
Survival SVM	0.688 (0.560-0.782)	0.688 (0.534-0.802)	0.694 (0.568-0.796)	0.714 (0.585-0.858)	0.057 (0.041-0.071)
CoxBoost	0.749 (0.671-0.822)	0.746 (0.640-0.855)	0.749 (0.647-0.838)	0.740 (0.587-0.843)	0.051 (0.032-0.069)
LightGBM	0.741 (0.655-0.823)	0.766 (0.663-0.830)	0.756 (0.654-0.836)	0.732 (0.624-0.862)	0.063 (0.050-0.072)
XGBoost (AFT)	0.750 (0.654-0.843)	0.791 (0.694-0.887)	0.762 (0.661-0.856)	0.768 (0.646-0.871)	0.055 (0.042-0.071)

a Model discrimination: Harrell’s C-index + time-dependent area under the curve; overall prediction error: integrated Brier score.

b AUC: area under the curve.

c RSF: random survival forest.

d SVM: support vector machine.

e AFT: accelerated failure time.

In the internal validation cohort, a slight attenuation in predictive performance was observed across all models. Nevertheless, XGBoost (AFT) maintained competitive discrimination (C-index=0.784, 95% CI 0.727‐0.833) and stable time-dependent AUCs at 3, 5, and 8 years (0.828, 0.822, and 0.789, respectively) (Figure 2D-F). In the external validation cohort, the XGBoost (AFT) model consistently demonstrated superior predictive performance, achieving a C-index of 0.750 (95% CI 0.654‐0.843). Furthermore, the model exhibited robust discriminative ability over time, with AUC values of 0.791, 0.762, and 0.768 at 3, 5, and 8 years, respectively (Figure 2G–I). Regarding other performance metrics, the XGBoost (AFT) model demonstrated optimal performance in predictive accuracy (0.740) and clinical utility (sensitivity=0.784, NPV=0.700) (Table S3 in Multimedia Appendix 1).

Calibration curves were generated for the 3 models with the highest C-index values in the external validation cohort (Figure S3 in Multimedia Appendix 1). In the training cohort, the 3-, 5-, and 8-year calibration curves of XGBoost (AFT) closely aligned with the ideal 45 degree line, indicating good agreement between predicted and observed risks. Clinical utility was assessed using decision curve analysis (Figure S4 in Multimedia Appendix 1), which revealed that the predictive curves for all cohorts remained well above the 2 baseline strategies. Although the decision curves intersected, XGBoost (AFT) provided comparable or higher net benefit across a clinically relevant range of threshold probabilities, supporting its potential utility in practice.

To assess the incremental predictive performance of the XGBoost (AFT) ML model relative to the traditional Cox proportional hazards regression model, we calculated the net reclassification improvement (NRI) and integrated discrimination improvement (IDI) at 3, 5, and 8 years of follow-up (Table S4 in Multimedia Appendix 1). For 3-year survival prediction, the XGBoost (AFT) model yielded an NRI of 0.338 (95% CI 0.180‐0.492, *P*=.01) and an IDI of 0.132 (95% CI 0.074‐0.188, *P*<.001). At 5 years, the corresponding values were an NRI of 0.311 (95% CI 0.172‐0.458, *P*=.01) and an IDI of 0.166 (95% CI 0.095‐0.235, *P*<.001). Similarly, for 8-year survival prediction, the XGBoost (AFT) model demonstrated a significant improvement over the Cox model, with an NRI of 0.265 (95% CI 0.113‐0.452, *P*=.02) and an IDI of 0.187 (95% CI 0.095‐0.281, *P*<.001). Across all evaluated time horizons, both NRI and IDI were statistically significant, indicating that the XGBoost (AFT) model achieved superior risk stratification and predictive discrimination compared with the conventional Cox model.

Overall, XGBoost (AFT) achieved the highest C-index among the evaluated models while maintaining competitive performance on other evaluation metrics across the cohorts. Considering its discriminative performance, predictive accuracy, and SHAP-based interpretability, XGBoost (AFT) was selected for subsequent interpretability analysis.

### Model Interpretability

To further elucidate the interpretability of the XGBoost (AFT) model, we used SHAP to quantify the contribution of each clinical feature to the model’s predictions of breast cancer DFS.

The SHAP summary plot (Figure 3A) illustrates the global importance of variables ranked according to their mean absolute SHAP values. pT stage emerged as the most influential feature, with a mean absolute SHAP value of 0.460, followed by endocrine therapy, LNR, pN stage, Ki-67, and chemotherapy (Table S5 in Multimedia Appendix 1). These findings indicate that pathological staging, particularly lymph node–related variables, together with treatment-related and molecular features, contributed substantially to the model’s DFS predictions.

**Figure 3.**
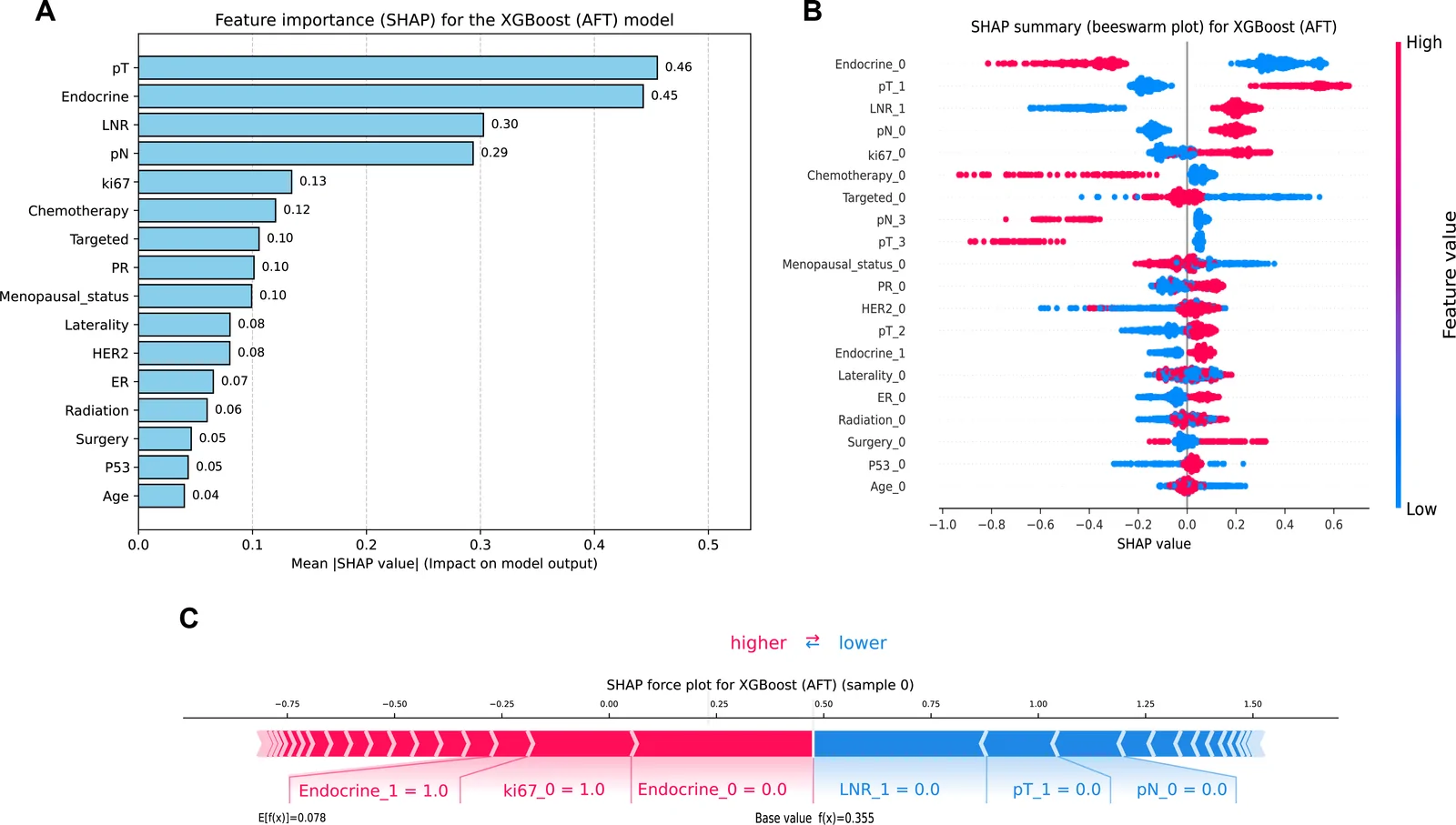
SHAP-based interpretation of the XGBoost (AFT) model. (A) Feature importance ranking based on SHAP values, illustrating the relative contributions of individual variables to the prediction of disease-free survival (DFS) in patients with breast cancer. (B) SHAP summary (beeswarm) plot. Positive SHAP values (right) indicate an increased risk of DFS, whereas negative SHAP values (left) indicate a reduced risk. Feature values are color-coded from low (blue) to high (red), demonstrating how different value ranges influence the model’s predictions. (C) SHAP force plot. The base value (*f*(*x*)=0.078) represents the average model prediction across all samples. Each colored bar represents a feature’s contribution to the final prediction for this sample. Red bars indicate features that push the prediction higher (toward a worse prognosis) relative to the base value. Blue bars indicate features that push the prediction lower (toward a better prognosis) relative to the base value. AFT: accelerated failure time; ER: estrogen receptor; HER2: human epidermal growth factor receptor 2; LNR: lymph node ratio; PR: progesterone receptor; SHAP: Shapley additive explanations; XGBoost: extreme gradient boosting.

As shown in Figure 3B, the SHAP beeswarm plot illustrates the direction and magnitude of feature contributions to the model predictions across different variable levels. Red dots representing N0 status and a low LNR (LNR<0.20) were predominantly distributed on the left, indicating contributions toward lower model-predicted risk, whereas those representing N3 disease and a high LNR (LNR>0.65) were mainly concentrated on the right, indicating contributions toward higher model-predicted risk. Similarly, endocrine therapy was characterized by predominantly leftward SHAP values, indicating a contribution toward lower model-predicted risk within the model. Red dots for T1 tumors were predominantly clustered on the left, whereas those for T3 tumors were mainly distributed on the right, indicating greater contributions toward higher model-predicted risk with increasing tumor stage.

As shown in Figure 3C, the SHAP force plot illustrates individual-level feature contributions to the XGBoost (AFT) model prediction for a randomly selected patient. The baseline model output was 0.078, whereas the final model output for this patient was 0.355, reflecting the combined contributions of the individual features. Thus, the difference between the patient-level prediction and the baseline output represents the aggregate contribution of the included features to the model prediction. Importantly, SHAP values describe feature contributions to model predictions and should not be interpreted as causal effects on DFS.

Overall, SHAP analysis provided global and individual-level explanations of the XGBoost (AFT) model predictions and improved the transparency and interpretability of the model. The observed contribution patterns were also broadly consistent with established clinical knowledge in breast oncology.

### Development of DFS Prediction Composite Index

SHAP feature importance analysis of the preliminary XGBoost (AFT) model showed comparable contributions of LNR and pN to model predictions. To further evaluate the robustness of the model to the inclusion of these variables, 2 additional XGBoost (AFT) models were constructed by separately excluding LNR or pN. Neither model showed a significant difference in predictive performance compared with the initial model containing both variables (Table S6 in Multimedia Appendix 1). Given their similar contributions and the comparable predictive performance observed in the sensitivity analyses, pN was retained because of its established role in TNM staging and greater clinical applicability, whereas LNR was excluded to improve model parsimony and facilitate clinical implementation.

The DFS score model demonstrated good discriminative ability across the training, internal validation, and external validation cohorts, with C-indices of 0.735, 0.725, and 0.715, respectively. The time-dependent AUC values at 3, 5, and 8 years consistently exceeded 0.70. In comparison, the traditional TNM staging system had a C-index of 0.618, with time-dependent AUC values of 0.625, 0.593, and 0.614 at 3, 5, and 8 years, respectively (Table 4).

**Table 4.** The efficacy of the disease-free survival risk score and tumor, node, metastasis stage in the training, internal validation, and external validation cohort^a^.

Cohorts and model	Harrell’s C-index (95% CI)	3-year AUC^b^ (95% CI)	5-year AUC (95% CI)	8-year AUC (95% CI)	Integrated Brier score (95% CI)
Training cohort (n=861)					
Risk score	0.735 (0.697-0.772)	0.751 (0.697-0.806)	0.762 (0.717-0.806)	0.728 (0.650-0.803)	0.069 (0.059-0.080)
TNM^c^	0.623 (0.587-0.655)	0.643 (0.597-0.685)	0.683 (0.600-0.761)	0.641 (0.600-0.680)	0.079 (0.069-0.091)
Internal validation cohort (n=370)					
Risk score	0.725 (0.666-0.783)	0.761 (0.672-0.842)	0.750 (0.691-0.806)	0.745 (0.684-0.804)	0.063 (0.048-0.077)
TNM	0.614 (0.559-0.668)	0.621 (0.544-0.696)	0.625 (0.562-0.689)	0.707 (0.548-0.814)	0.071 (0.055-0.087)
External validation cohort (n=304)					
Risk score	0.715 (0.604-0.815)	0.762 (0.650-0.862)	0.731 (0.671-0.806)	0.722 (0.657-0.781)	0.058 (0.041-0.076)
TNM	0.618 (0.536-0.714)	0.625 (0.524-0.731)	0.593 (0.512-0.700)	0.614 (0.506-0.715)	0.063 (0.043-0.081)

a Risk Score = (0.755 × pN_1) + (1.239 × pN_2) + (1.587 × pN_3) + (0.651 × pT_2) + (1.340 × pT_3) + (1.698 × pT_4) + (−0.739 × Endocrine_Yes) + (0.192 × Ki67_High) + (−0.961 × Chemotherapy_Yes).

b AUC: area under the curve.

c TNM: tumor, node, metastasis.

Patients were stratified into low- and high-risk groups using the median risk score as the cutoff. DFS differed significantly between the 2 groups (log-rank test, *P*<.001), with a higher model-predicted risk corresponding to shorter DFS (HR 5.44, 95% CI 4.04‐7.31; *P*<.001). A significant trend toward shorter DFS was also observed across increasing risk levels (trend test, *P*<.001). The 5-year DFS rates were 91.7% and 60.2% in the low- and high-risk groups, respectively. Kaplan-Meier curves showed early separation between the risk groups, and the curves remained separated during follow-up (Figure 4).

**Figure 4.**
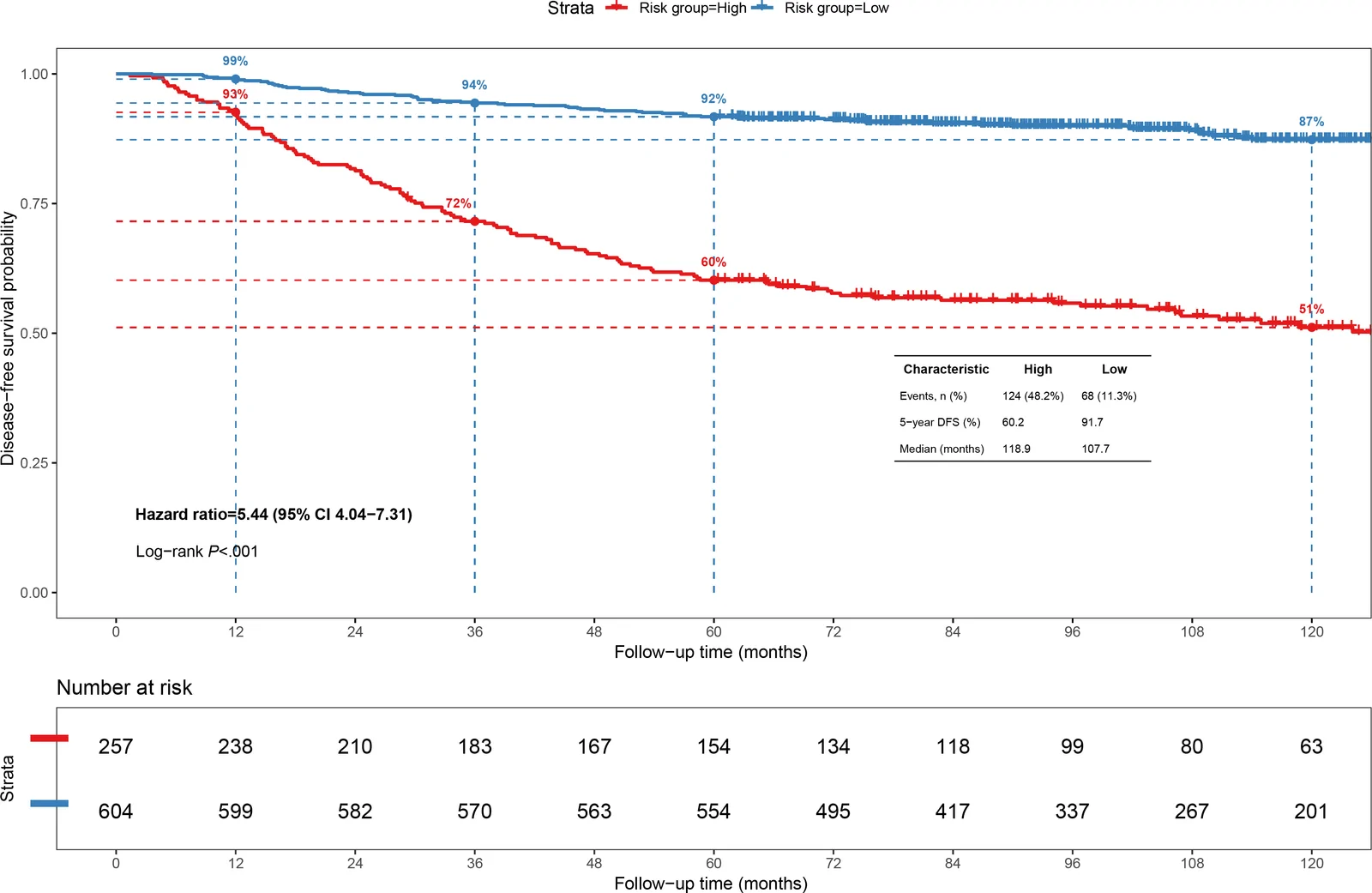
Kaplan-Meier curves for the composite disease-free survival (DFS) score model.

## Discussion

### Principal Findings

Based on a clinical cohort of patients with breast cancer from our institution, this study evaluated 5 ML algorithms, with Cox regression as a conventional statistical reference, to develop a model for postoperative DFS prediction. SHAP analysis was used to characterize the contributions of individual features to model predictions and improve model interpretability. A composite DFS score was further developed for individualized prognostic risk stratification [18-20].

Our model was constructed using a clinical cohort characterized by long-term follow-up, with a median DFS of 109 (IQR 85.0-134.2) months (approximately 9.1 y) among patients. This duration significantly exceeds that of many retrospective studies focusing on 5-year survival rates, highlighting a key advantage of our predictive model. An extensive body of research indicates that 1 to 3 years after surgery represents the peak period for breast cancer recurrence, and the risk of recurrence increases gradually after 5 years, while extending adjuvant therapy beyond this period can improve patient outcomes [21-23]. Moreover, high-risk patients exhibit a higher risk of recurrence across all time intervals, suggesting that the period beyond 5 years after surgery is also a critical phase for disease progression [24,25]. The long-term follow-up of this cohort not only supports the assessment of the ML model’s stability in long-term prediction [26] but also enables its evaluation using data from TCGA. Recent studies have further emphasized the importance of dynamic biomarkers and long-term prognostic assessment in breast cancer, including changes in hormone receptor and HER2 status after neoadjuvant chemotherapy [27]. In the present study, the 5-year outcome was considered the primary time horizon for evaluating model performance because of its clinical relevance and the relatively more mature follow-up available at this time point. The 8-year analysis was additionally performed to explore the potential long-term applicability of the model; however, because of the limited long-term follow-up in the TCGA external validation cohort, the 8-year results should be interpreted as exploratory findings rather than confirmatory evidence.

Our results demonstrated that the XGBoost (AFT) model exhibited the best performance among the 5 ML models in the development, internal validation, and external validation sets. In the development set, the C-index for the XGBoost (AFT) model was 0.808. The AUC values for predicting 3-, 5-, and 8-year DFS were 0.833, 0.840, and 0.824, respectively. Notably, the 5-year AUC was 0.840 in the development cohort and remained 0.762 in the external validation cohort, supporting the model’s ability to provide meaningful medium-term DFS predictions across different populations. Furthermore, this model demonstrated optimal performance in both the external and internal validation sets, indicating its predictive stability. Although the 8-year AUC was also evaluated, this result should be interpreted cautiously because the TCGA external validation cohort had limited long-term follow-up. Therefore, the 8-year findings are considered exploratory and should not be interpreted as definitive evidence of sustained long-term predictive performance. In contrast, the traditional TNM staging model had a C-index of 0.618 in the external validation cohort, with corresponding 3-, 5-, and 8-year AUC values of 0.625, 0.593, and 0.614, respectively. These results indicate that the predictive model established in this study outperformed the traditional TNM staging model for predicting DFS. Compared with the Cox model, the XGBoost (AFT) model demonstrated more pronounced improvements in the NRI and IDI, thereby potentially enhancing its clinical utility. These findings are consistent with recent studies demonstrating the potential of ML and SHAP-based approaches to improve prognostic and treatment-response prediction in breast cancer [28,29].

The traditional TNM staging system is the clinical gold standard for assessing breast cancer prognosis. However, it is limited to anatomical features such as tumor size, lymph node involvement, and distant metastasis. This limitation of anatomical staging overlooks the prognostic impact of clinicopathological factors, including patient age, histology, molecular subtype, and adjuvant therapy, thereby limiting its ability to precisely evaluate the influence of clinical heterogeneity and continuous variables on prognosis [30]. In contrast to the linear and proportional hazards assumptions of traditional TNM staging models, ML algorithms can effectively capture nonlinear relationships between features and prognostic end points, as well as complex interactions among features [31]. This may explain the significantly superior predictive performance of the ML models compared with the traditional model in this study. Previous studies have often focused on constructing and validating single-algorithm models due to limitations such as algorithm selection bias and insufficient generalizability [32]. This study systematically compared the predictive performance of 5 ML algorithms, with Cox regression included as a conventional statistical reference, to identify the model with the best overall performance for postoperative DFS prediction in our cohort. Model development and internal validation were conducted using a real-world clinical cohort from our institution, whereas external validation was performed using the independent TCGA cohort. This validation strategy provided an assessment of the model’s generalizability to an independent patient population and data source. Recent studies have similarly explored ML-based prognostic models using TCGA and Gene Expression Omnibus datasets, highlighting the potential of integrating clinical and molecular information for individualized risk prediction [33-35]. Nevertheless, the external validation in the present study should be considered supportive evidence of model generalizability rather than a substitute for prospective multicenter validation.

Although ML models can achieve strong predictive performance, their complex decision-making processes may be difficult for clinicians to interpret, which can hinder their translation into clinical practice [36]. In this study, SHAP analysis was applied to the optimal XGBoost (AFT) model to provide both global and individual-level interpretations of model predictions. The results identified pT, endocrine therapy, pN, LNR, Ki-67, and chemotherapy as among the most important contributors to the model predictions. Consistent with previous studies, higher pT, LNR, pN, and Ki-67 expression, as well as the absence of endocrine therapy and chemotherapy, contributed to higher model-predicted risk [37,38]. Importantly, LNR and pN showed comparable contributions to model predictions. To further evaluate the robustness of their inclusion, sensitivity analyses were performed by separately excluding LNR or pN from the XGBoost (AFT) model. Neither model showed a significant difference in predictive performance compared with the model containing both variables (Table S6 in Multimedia Appendix 1). Given their comparable predictive performance, pN was retained in the final model because of its established role in TNM staging and broader clinical applicability, whereas LNR was excluded to improve model parsimony.

The SHAP force plot further illustrated how individual features contributed to the prediction for a randomly selected patient relative to the model’s baseline output. This individual-level explanation provides a transparent representation of how the included features collectively shaped the model prediction and may facilitate the interpretation of patient-specific risk estimates. Overall, SHAP analysis provided both global and individual-level explanations of the XGBoost (AFT) model predictions, demonstrating that the model’s decision patterns were broadly consistent with established clinical knowledge in breast oncology while improving the transparency and interpretability of the model. This interpretable framework may support individualized prognostic risk assessment and risk stratification in patients with breast cancer.

Accurate prognostic risk stratification is important for the individualized management and follow-up of patients with breast cancer [39]. However, the TNM staging system has limited ability to capture prognostic heterogeneity among patients with the same stage and does not incorporate multiple clinical, pathological, and treatment-related factors [40]. In this study, a composite DFS risk score was developed by integrating variables identified through SHAP-based feature importance analysis of the optimal ML model with independent prognostic factors identified by multivariable Cox regression. The risk score was calculated as a linear combination of the selected variables, with the corresponding regression coefficients used as variable weights [41]. The resulting DFS score demonstrated better predictive performance than the traditional TNM staging system. Using the median risk score as the cutoff, patients were stratified into low- and high-risk groups, with significantly different DFS between the groups. These findings indicate that the score can effectively distinguish patients according to their model-predicted prognostic risk and may provide a practical approach to postoperative risk stratification. Its potential clinical value lies primarily in identifying patients with different levels of postoperative recurrence risk and supporting individualized surveillance and further clinical assessment. However, because this was a retrospective observational study, the risk score should not be interpreted as a tool for directly recommending treatment intensification or de-escalation. In particular, the treatment variables represented postoperative treatments actually received, and their predictive importance should therefore be interpreted as prognostic information within the model rather than as causal treatment effects.

Recent research has further expanded the application of ML in breast cancer prognosis by incorporating dynamic biomarkers, multiomics data, and multimodal information. Changes in hormone receptor and HER2 status following neoadjuvant chemotherapy have been associated with subsequent oncological outcomes, suggesting that dynamic biomarker assessment may provide prognostic information beyond baseline measurements [27]. Recent studies have similarly suggested that ML-derived prognostic signatures may provide complementary information to conventional clinicopathological staging by integrating multiple dimensions of disease biology. Importantly, emerging studies have begun to extend this approach beyond conventional clinical variables by integrating molecular, multiomics, pathological, and imaging information [42]. For example, multiomics and ML studies have identified potential prognostic biomarkers and biological pathways, including PLA2G4A- and APOO-related molecular characteristics [34,35]. In addition, recent studies have developed prognostic models using multiple ML algorithms and immune- or metabolism-related molecular features [33]. Multimodal approaches integrating clinical information with pathology-derived features, deep learning features, and MRI radiomics have also shown promising performance for survival prediction in breast cancer [43]. These findings suggest that future iterations of our model could potentially incorporate longitudinal blood biomarkers, digital pathology, radiomics, and genomic or transcriptomic information while retaining SHAP-based interpretability. Explainable AI may provide a useful framework for integrating heterogeneous data sources and presenting complex model predictions in a clinically interpretable manner [29]. Other recent real-world studies, including pharmacovigilance analyses of breast cancer therapies, have further highlighted the value of complementary real-world evidence for evaluating treatment-related outcomes and safety [44]. Although these outcomes were beyond the scope of the present DFS model, they underscore the importance of evaluating predictive models within real-world clinical contexts. Methodological studies in other cancers have likewise demonstrated the potential of integrating ML with immune, transcriptomic, and single-cell information [45]; however, these findings should not be directly extrapolated to breast cancer.

### Limitations

This study has several limitations. First, the retrospective single-center design and use of the TCGA cohort for external validation may introduce selection and information biases, whereas differences in patient characteristics and treatment patterns may affect model performance. Although external validation provided supportive evidence of generalizability, prospective multicenter validation is warranted. We are also collaborating with other institutions to collect independent clinical data, including blood-based laboratory indicators and additional clinicopathological variables, for future validation. Second, the model mainly incorporated conventional clinicopathological and treatment-related variables and did not include genomic, transcriptomic, multiomics, or radiomic information, which may provide additional prognostic information. Third, the TCGA cohort had limited long-term follow-up. Thus, although 8-year DFS prediction was evaluated, these results should be considered exploratory, and the 5-year results should be regarded as the principal evidence of model performance. Fourth, treatment variables represented postoperative treatments actually received, and their predictive importance should be interpreted as prognostic information within the model rather than as causal treatment effects. Fifth, the institutional cohort spanned the transition from the seventh to the eighth edition of the American Joint Committee on Cancer staging system during 2012 to 2020. Although the anatomic pT and pN categories used in this study did not undergo substantive changes between the 2 editions and were therefore comparable across the study period, minor staging-related heterogeneity cannot be completely excluded. In addition, Ki-67 was assessed by immunohistochemistry in the institutional cohort, and potential interobserver and methodological heterogeneity in Ki-67 assessment may have affected its classification. In the TCGA-BRCA cohort, standardized immunohistochemistry measurements were unavailable; therefore, MKI67 and TP53 mRNA expression were used as transcriptomic surrogate measures for external validation. These transcriptomic measures are not directly equivalent to immunohistochemistry-derived Ki-67 and p53 protein expression, particularly because TP53 transcript abundance does not directly reflect p53 protein accumulation. Although sensitivity analysis excluding TP53 showed essentially unchanged external validation performance and SHAP contribution patterns, this transcriptomic-protein-level discrepancy should be considered when interpreting the external validation results. Finally, although SHAP enhanced model interpretability, prospective clinical evaluation is needed to establish the clinical utility of the model.

### Conclusions

In summary, this study developed and externally validated an interpretable ML model for postoperative DFS prediction in patients with breast cancer. The XGBoost (AFT) model demonstrated better predictive performance than conventional TNM staging, whereas SHAP analysis enhanced model interpretability. The derived risk score enabled prognostic risk stratification and provided a quantitative approach to individualized risk assessment and postoperative follow-up management. Further prospective multicenter validation is warranted before broader clinical application.

## Supplementary material

10.2196/101633 Multimedia Appendix 1 Supplementary figures and tables presenting detailed patient characteristics, model performance, and validation results for the machine learning–based disease-free survival prediction models.

10.2196/101633 Checklist 1 STROBE checklist.

## Abbreviations

AFT: accelerated failure time
AUC: area under the curve
DFS: disease-free survival
ER: estrogen receptor
HER2: human epidermal growth factor receptor 2
HR: hazard ratio
IDI: integrated discrimination improvement
LightGBM: light gradient boosting machine
LNR: lymph node ratio
ML: machine learning
NRI: net reclassification improvement
SHAP: Shapley additive explanations
TCGA: The Cancer Genome Atlas
TNM: tumor, node, metastasis
XGBoost: extreme gradient boosting
