# Supplementary material for: Machine Learning–Based Prediction of Disease-Free Survival in Breast Cancer Incorporating Clinical and Treatment Information: Retrospective Cohort Study With External Validation
Source: JMIR Med Inform. 2026 Nov 13;14:e101633. doi: 10.2196/101633 (PMC13766663; doi:10.2196/101633)
Supplement: Supplementary file 1 [file medinform-v14-e101633-s002.pdf]

STROBE Statement—Checklist of items that should be included in reports of ***cohort studies***

	Item No	Recommendation	Page No
**Title and abstract**	1	(*a*) Indicate the study's design with a commonly used term in the title or the abstract	1-3
		(*b*) Provide in the abstract an informative and balanced summary of what was done and what was found	26-61
**Introduction**			
Background/rationale	2	Explain the scientific background and rationale for the investigation being reported	70-108
Objectives	3	State specific objectives, including any prespecified hypotheses	109-117
**Methods**			
Study design	4	Present key elements of study design early in the paper	120-126
Setting	5	Describe the setting, locations, and relevant dates, including periods of recruitment, exposure, follow-up, and data collection	127-161
Participants	6	(*a*) Give the eligibility criteria, and the sources and methods of selection of participants. Describe methods of follow-up	122-139
		(*b*) For matched studies, give matching criteria and number of exposed and unexposed	NA
Variables	7	Clearly define all outcomes, exposures, predictors, potential confounders, and effect modifiers. Give diagnostic criteria, if applicable	162-172
Data sources/ measurement	8*	For each variable of interest, give sources of data and details of methods of assessment (measurement). Describe comparability of assessment methods if there is more than one group	134-161
Bias	9	Describe any efforts to address potential sources of bias	140-145
Study size	10	Explain how the study size was arrived at	120-133
Quantitative variables	11	Explain how quantitative variables were handled in the analyses. If applicable, describe which groupings were chosen and why	146-172
Statistical methods	12	(*a*) Describe all statistical methods, including those used to control for confounding	176-189
		(*b*) Describe any methods used to examine subgroups and interactions	NA
		(*c*) Explain how missing data were addressed	140-145
		(*d*) If applicable, explain how loss to follow-up was addressed	122-126
		(*e*) Describe any sensitivity analyses	187-189
**Results**			
Participants	13*	(a) Report numbers of individuals at each stage of study—eg numbers potentially eligible, examined for eligibility, confirmed eligible, included in the study, completing follow-up, and analysed	238-261
		(b) Give reasons for non-participation at each stage	244-261
		(c) Consider use of a flow diagram	Figure 1
Descriptive data	14*	(a) Give characteristics of study participants (eg demographic, clinical, social) and information on exposures and potential confounders	Table 1 244-261
		(b) Indicate number of participants with missing data for each variable of interest	140-145
		(c) Summarise follow-up time (eg, average and total amount)	238-243
Outcome data	15*	Report numbers of outcome events or summary measures over time	265-285

Main results	16	(*a*) Give unadjusted estimates and, if applicable, confounder-adjusted estimates and their precision (eg, 95% confidence interval). Make clear which confounders were adjusted for and why they were included	286-324
		(*b*) Report category boundaries when continuous variables were categorized	NA
		(*c*) If relevant, consider translating estimates of relative risk into absolute risk for a meaningful time period	401-408
Other analyses	17	Report other analyses done—eg analyses of subgroups and interactions, and sensitivity analyses	385-408
**Discussion**			
Key results	18	Summarise key results with reference to study objectives	415-463
Limitations	19	Discuss limitations of the study, taking into account sources of potential bias or imprecision. Discuss both direction and magnitude of any potential bias	570-602
Interpretation	20	Give a cautious overall interpretation of results considering objectives, limitations, multiplicity of analyses, results from similar studies, and other relevant evidence	415-470
Generalisability	21	Discuss the generalisability (external validity) of the study results	603-609
**Other information**			
Funding	22	Give the source of funding and the role of the funders for the present study and, if applicable, for the original study on which the present article is based	613-616

*Give information separately for exposed and unexposed groups.

Note: An Explanation and Elaboration article discusses each checklist item and gives methodological background and published examples of transparent reporting. The STROBE checklist is best used in conjunction with this article (freely available on the Web sites of PLoS Medicine at http://www.plosmedicine.org/, Annals of Internal Medicine at http://www.annals.org/, and Epidemiology at http://www.epidem.com/). Information on the STROBE Initiative is available at http://www.strobe-statement.org.
