# Supplementary material for: Machine Learning–Based Prediction of Disease-Free Survival in Breast Cancer Incorporating Clinical and Treatment Information: Retrospective Cohort Study With External Validation
Source: JMIR Med Inform. 2026 Nov 13;14:e101633. doi: 10.2196/101633 (PMC13766663; doi:10.2196/101633)
Supplement: Supplementary file 2 [file medinform-v14-e101633-s001.docx]

**Supplementary Table 1** The optimal hyper-parameters and metrics of machine learning models

| **Model** | **Hyperparameter** |
| --- | --- |
| RSF | n_estimators=500, min_samples_split=10, min_samples_leaf=15, max_features="sqrt",n_jobs=-1 |
| Survival SVM | alpha=1.0, rank_ratio=1.0, max_iter=5000 |
| CoxBoost | loss="coxph", learning_rate=0.05, n_estimators=800 |
| LightGBM | n_estimators=800, learning_rate=0.03, num_leaves=31, min_child_samples=20, subsample=0.8, colsample_bytree=0.8, reg_lambda=1.0, |
| XGBoost (AFT) | learning_rate: 0.03, max_depth: 2, subsample: 0.7, colsample_bytree: 0.8 |

**Supplementary Table 2** VIFs of Composite Recurrence Score model

| Variables | GVIF^(1/(2*Df)) |
| --- | --- |
| Age | 1.293 |
| Surgery | 1.069 |
| Menopausal status | 1.226 |
| ER | 2.067 |
| PR | 1.744 |
| HER2 | 2.132 |
| Laterality | 1.026 |
| Chemotherapy | 1.169 |
| Endocrine | 1.886 |
| Targeted therapy | 2.082 |
| Radiation therapy | 1.221 |
| pT | 1.065 |
| pN | 1.058 |
| LNR | 1.807 |
| Ki-67 expression | 1.156 |
| P53 expression | 1.058 |

Abbreviation: ER: estrogen receptor; PR: progesterone receptor; HER2: human epidermal growth factor receptor; LNR: Lymph Node Ratio.

**Supplementary Table 3** Evaluation of the Performance of Six Models in Predicting Breast Cancer DFS.

| Cohorts | Models | AUC (95%CI) | Sensitivity | Specificity | Accuracy | PPV | NPV | F1-score | Recall |
| --- | --- | --- | --- | --- | --- | --- | --- | --- | --- |
| Training cohort  (n = 861) | Cox regression | 0.739  (0.694, 0.771) | 0.712 | 0.830 | 0.769 | 0.581 | 0.847 | 0.640 | 0.712 |
|  | RSF | 0.771  (0.732, 0.800) | 0.718 | 0.832 | 0.773 | 0.587 | 0.850 | 0.646 | 0.718 |
|  | Survival SVM | 0.750  (0.719, 0.782) | 0.771 | 0.780 | 0.750 | 0.540 | 0.860 | 0.635 | 0.771 |
|  | CoxBoost | 0.794  (0.769, 0.827) | 0.747 | 0.853 | 0.795 | 0.639 | 0.862 | 0.689 | 0.747 |
|  | LightGBM | 0.805  (0.781, 0.839) | 0.782 | 0.846 | 0.764 | 0.640 | 0.874 | 0.703 | 0.782 |
|  | XGBoost (AFT) | 0.808  (0.771, 0.832) | 0.806 | 0.825 | 0.800 | 0.659 | 0.879 | 0.725 | 0.806 |
| Internal validation cohort  (n = 370) | Cox regression | 0.754  (0.701, 0.794) | 0.679 | 0.806 | 0.736 | 0.571 | 0.811 | 0.620 | 0.679 |
|  | RSF | 0.752  (0.690, 0.792) | 0.705 | 0.771 | 0.720 | 0.541 | 0.814 | 0.612 | 0.705 |
|  | Survival SVM | 0.755  (0.696, 0.799) | 0.745 | 0.712 | 0.691 | 0.500 | 0.818 | 0.598 | 0.745 |
|  | CoxBoost | 0.765  (0.713, 0.808) | 0.705 | 0.818 | 0.752 | 0.627 | 0.822 | 0.660 | 0.705 |
|  | LightGBM | 0.780  (0.742, 0.814) | 0.745 | 0.818 | 0.718 | 0.633 | 0.837 | 0.682 | 0.745 |
|  | XGBoost (AFT) | 0.784  (0.727, 0.833) | 0.784 | 0.765 | 0.792 | 0.665 | 0.844 | 0.724 | 0.784 |
| External validation cohort  (n = 304) | Cox regression | 0.707  (0.584, 0.819) | 0.648 | 0.821 | 0.708 | 0.773 | 0.622 | 0.705 | 0.648 |
|  | RSF | 0.728  (0.629, 0.819) | 0.616 | 0.821 | 0.701 | 0.762 | 0.605 | 0.681 | 0.616 |
|  | Survival SVM | 0.688  (0.560, 0.782) | 0.648 | 0.857 | 0.715 | 0.810 | 0.632 | 0.720 | 0.648 |
|  | CoxBoost | 0.749  (0.671, 0.822 | 0.584 | 0.786 | 0.717 | 0.714 | 0.579 | 0.642 | 0.584 |
|  | LightGBM | 0.741  (0.655, 0.823) | 0.584 | 0.893 | 0.701 | 0.833 | 0.610 | 0.687 | 0.584 |
|  | XGBoost (AFT) | 0.750  (0.654, 0.843) | 0.784 | 0.857 | 0.740 | 0.789 | 0.700 | 0.786 | 0.784 |

PPV, positive predictive value; NPV, negative predictive value.

**Supplementary Table 4** Incremental Predictive Value of the XGBoost (AFT) Machine Learning Model Compared with the Cox Model

| Follow-up Duration | NRI (95%CI) | *P* | IDI (95%CI) | *P* |
| --- | --- | --- | --- | --- |
| 3-year | 0.338 (0.180, 0.492) | 0.010 | 0.132 (0.074, 0.188) | < 0.001 |
| 5-year | 0.311 (0.172, 0.458) | 0.013 | 0.166 (0.095, 0.235) | < 0.001 |
| 8-year | 0.265 (0.113, 0.452) | 0.018 | 0.187 (0.095, 0.281) | < 0.001 |

IDI, Integrated Discrimination Improvement; NRI, Net reclassification improvement.

**Supplementary Table 5** Mean absolute SHAP values of XGBoost (AFT)

| **Variable** | **Mean \|SHAP value\|** |
| --- | --- |
| pT | 0.460 |
| Endocrine therapy | 0.447 |
| Lymph Node Ratio (LNR) | 0.297 |
| pN | 0.289 |
| Ki-67 expression | 0.133 |
| Chemotherapy | 0.121 |
| PR | 0.104 |
| Targeted therapy | 0.104 |
| Menopausal status | 0.102 |
| Laterality | 0.079 |
| HER2 | 0.078 |
| ER | 0.065 |
| Radiation therapy | 0.062 |
| Surgery | 0.046 |
| P53 expression | 0.045 |
| Age | 0.043 |

**Supplementary Table 6** The efficacy of the XGBoost (AFT) model after excluding LNR, pN and P53 respectively.

| Cohorts | Models | Harrell’s C-index (95%CI) | 3-year AUC (95%CI) | 5-year AUC (95%CI) | 8-year AUC (95%CI) | Integrated Brier score (95%CI) |
| --- | --- | --- | --- | --- | --- | --- |
| Training cohort  (n = 861) | Model excluding LNR | 0.822  (0.785 - 0.846) | 0.856  (0.813 - 0.887) | 0.859  (0.825 - 0.882) | 0.834  (0.785 - 0.863) | 0.107  (0.096-0.118) |
|  | Model excluding pN | 0.812  (0.783 - 0.842) | 0.834  (0.742 - 0.892) | 0.834  (0.791 - 0.872) | 0.818  (0.805 - 0.872) | 0.106  (0.095-0.117) |
|  | Model excluding P53 | 0.805  (0.774, 0.836) | 0.832  (0.787, 0.874) | 0.838  (0.802, 0.873) | 0.823  (0.787, 0.859) | 0.103  (0.093, 0.114) |
| Internal validation cohort  (n = 370) | Model excluding LNR | 0.774  (0.725 - 0.819) | 0.806  (0.749 - 0.926) | 0.834  (0.770 - 0.893) | 0.808  (0.739 - 0.829) | 0.105  (0.089-0.121) |
|  | Model excluding pN | 0.768  (0.718 - 0.814) | 0.789  (0.720 - 0.930) | 0.809  (0.773 - 0.896) | 0.793  (0.759 - 0.868) | 0.103  (0.088-0.120) |
|  | Model excluding P53 | 0.768  (0.718 - 0.814) | 0.820  (0.747, 0.880) | 0.820  (0.761, 0.870) | 0.785  (0.722, 0.844) | 0.107  (0.092, 0.125) |
| External validation cohort  (n = 304) | Model excluding LNR | 0.745  (0.659 - 0.826) | 0.787  (0.671, 0.870) | 0.765  (0.678 - 0.847) | 0.785  (0.683 - 0.822) | 0.055  (0.041-0.070) |
|  | Model excluding pN | 0.744  (0.663 - 0.818) | 0.769  (0.569 - 0.897) | 0.757  (0.657 - 0.848) | 0.760  (0.648 - 0.832) | 0.054  (0.040-0.071) |
|  | Model excluding P53 | 0.749  (0.660, 0.834) | 0.788  (0.688, 0.878) | 0.760  (0.658, 0.853) | 0.767  (0.630, 0.882) | 0.054  (0.040, 0.073) |

**Supplementary Table 7** Relative contribution of variables based on mean absolute SHAP values.

| **Variable** | **Mean \|SHAP value\|** | **Contribution (%)** |
| --- | --- | --- |
| pT | 0.460 | 21.12% |
| Endocrine therapy | 0.447 | 20.52% |
| pN | 0.289 | 13.27% |
| Ki67 expression | 0.133 | 6.11% |
| Chemotherapy | 0.121 | 5.56% |
| PR | 0.104 | 4.78% |
| Targeted therapy | 0.104 | 4.78% |
| Menopausal status | 0.102 | 4.68% |
| Laterality | 0.079 | 3.63% |
| HER2 | 0.078 | 3.58% |
| ER | 0.065 | 2.98% |
| Radiation therapy | 0.062 | 2.85% |
| Surgery | 0.046 | 2.11% |
| P53 expression | 0.045 | 2.07% |
| Age | 0.043 | 1.97% |

**Supplementary Figure 1** Correlation heatmap of all variables (Correlation analyses were conducted based on variable types: the polychoric correlation coefficient was used for pairs of ordinal categorical variables, while Cramér's V was employed for associations involving at least one nominal variable)

**Supplementary Figure 2** KM curve of variables with statistical significance in univariate Cox analysis. (A)Surgery; (B)Menopausal status; (C)Ki67; (D)pT; (E)pN; (F)LNR; (G)Endocrine therapy; (H)Radiation therapy; (I)ER.

**Supplementary Figure** 3 Calibration curves of the breast cancer recurrence prediction model. A–C: Calibration curves were used to assess the agreement between the predicted probabilities and the actual observed outcomes for each model in the development set. D–F: Calibration curves were used to assess the agreement between the predicted probabilities and the actual observed outcomes for each model in the internal validation set. G–I: Calibration curves were used to assess the agreement between the predicted probabilities and the actual observed outcomes for each model in the external validation set.

**Supplementary Figure** 4 Decision curve analysis (DCA) of the breast cancer recurrence prediction model.A–C: DCA was performed to evaluate the clinical net benefit provided by each model across various threshold probability ranges within the development set. D–F: DCA was performed to evaluate the clinical net benefit provided by each model across various threshold probability ranges within the internal validation set. G–I: DCA was performed to evaluate the clinical net benefit provided by each model across various threshold probability ranges within the TCGA validation set.
